# Gag reflex management in dental practice: a bibliometric analysis from 2000 to 2025

**DOI:** 10.3389/froh.2025.1666164

**Published:** 2025-09-24

**Authors:** Yena Gan, Jinwei Huang, Duoduo Li, He Xu, Sheng Han, He Zhu, Zening Wei, Zhigang Cai

**Affiliations:** 1 Department of Academic Research, International Research Center for Medicinal Administration, Peking University, Beijing, China; 2 Department of General Dentistry Ⅱ, Peking University School and Hospital of Stomatology, Beijing, China; 3 National Center for Stomatology & National Clinical Research Center for Oral Diseases & National Engineering Research Center of Oral Biomaterials and Digital Medical Devices, Beijing, China; 4 Department of Tuina and Pain, Dongzhimen Hospital, Beijing University of Chinese Medicine, Beijing, China; 5 Department of Pediatric Dentistry, Peking University School and Hospital of Stomatology, Beijing, China; 6 Institute of Medical Innovation and Research & Medical Research Center, Peking University Third Hospital, Beijing, China; 7 Department of Oral and Maxillofacial Surgery, Peking University School and Hospital of Stomatology, Beijing, China

**Keywords:** bibliometric analysis, gag reflex, treatment avoidance, acupuncture, dental treatment

## Abstract

**Background:**

The gag reflex is a common challenge in dental practice, often causing discomfort and leading to treatment avoidance, especially during procedures like taking dental impressions. Although both pharmacological and non-pharmacological treatments are utilized to manage the gag reflex, current evidence supporting the routine use of these interventions is limited, highlighting the need for further research. To optimize gag reflex management and identify future research trends through a bibliometric analysis.

**Methods:**

Data from eligible studies were extracted through a comprehensive search and various analyses, including descriptive bibliometric, citation, keyword, and thematic analyses. Group comparisons were made between Asian and non-Asian groups to assess the differences and trends between the two regions.

**Results:**

Between 2000 and 2025, 47 studies were published, with an average annual increase of 4.68%. Japan, Iran, India, the USA, and Turkey each contributed at least 10 studies, and studies from Lebanon had the highest citation frequency. The majority of studies were randomized controlled trials (46.81%), followed by case reports (46.81%) and case series (6.38%). Most studies (80.85%) focused on adults undergoing extractions (38.30%), restorations (31.91%), and dental impressions (14.89%). Interventions primarily targeted nausea and vomiting (74.47%) using pharmacological treatments such as lidocaine, propofol, and midazolam and non-pharmacological methods such as acupuncture, acupressure, and Transcutaneous Electrical Nerve Stimulation. Significant differences were observed between Asian and non-Asian groups in publication years, study designs, demographics, treatments, and clinical outcomes.

**Conclusions:**

Gag reflex management has advanced with pharmacological treatments for immediate relief and increased use of non-pharmacological methods such as acupuncture and laser stimulation, particularly in Japan, Turkey, and the USA. However, challenges such as small-scale studies, limited follow-ups, and underrepresentation of children and adolescents highlight the need for larger studies, standardized tools, and inclusive approaches for diverse patient groups.

**Systematic Review Registration:**

https://www.crd.york.ac.uk/PROSPERO/view/CRD420250650382, identifier (CRD420250650382).

## 1 Introduction

The gag reflex is a prevalent clinical challenge in dental practice that complicates taking dental impressions, endodontic treatments, restorations, and extractions (1). Discomfort caused by the gag reflex, such as nausea and vomiting, can adversely affect patient satisfaction and potentially lead to treatment avoidance. Approximately 8.2% of dental patients experience discomfort related to the gag reflex (2). Moreover, up to 20% of patients have been observed to avoid dental procedures because of the gag reflex (3). Various stimuli, including mechanical, auditory, olfactory, visual, and psychological factors, can trigger or exacerbate the gag reflex during dental treatment.

Gag reflex management involves both pharmacological and non-pharmacological interventions. Pharmacological approaches employ peripheral agents to reduce throat sensitivity (4–7) or centrally acting agents, such as antihistamines (8), sedatives (6, 9), and anticholinergics (10), to modulate the nervous system and decrease gag reflex intensity. For severe cases requiring more invasive treatments, intravenous sedation and local anesthetics are used (9, 11). Additionally, increased concentrations of nitrous oxide can control severe gag reflexes and help patients tolerate procedures such as dental radiographs (12). Non-pharmacological interventions include multiple techniques, including acupuncture (13–15), acupressure (15, 16), laser stimulation (17, 18), and earplugs (19). Notably, acupressure on the P6 (Pericardium 6, Neiguan) acupoint and low-level laser therapy have been shown to alleviate both the gag reflex and anxiety (20–22). Distraction techniques such as games have proven effective in reducing severity, particularly in children (23, 24). Combined interventions such as hypnopuncture (hypnosis and acupuncture) have also been explored to enhance patient comfort and manage symptoms during dental procedures (3, 5, 25).

However, current literature on this topic provides limited and low-quality evidence (26). Further studies are needed to comprehensively analyze the effectiveness of these interventions in the management of dental-related gag reflexes. This study employed bibliometric analysis to examine the trends, advancements, and future directions of gag reflex interventions during dental treatment with the aim of optimizing management strategies.

## 2 Materials and methods

A comprehensive literature search of titles or abstracts with the keywords and synonyms of “gag” and “dental” was performed across the Scopus, Web of Science Core Collection (WoSCC), PubMed, Embase, and the Cochrane Library databases in February 2025 (see Supplementary Additional file S1). The research protocol was registered in the International Prospective Register of Systematic Reviews (No. CRD420250650382). After removing duplicates, two investigators (J.H. and Y.G.) independently screened the publications for eligibility. The full texts were reviewed as needed, and any discrepancies were resolved by a third investigator (H.X.).

The PICOS framework (participant, intervention, comparator, outcome, and study design) was used to identify potentially eligible studies. The participants were patients experiencing difficulties during dental treatment due to the gag reflex or a history of gag reflex-induced nausea, vomiting, fear, and avoidance behaviors. Interventions aimed at alleviating gag reflex-related symptoms, including pharmacological treatments, acupressure, acupuncture, and hypnosis, were considered. The effectiveness of these interventions was also assessed. Eligible studies included randomized controlled trials (RCTs), cohort studies, case reports, and case series. Only English language studies published between 2000 and 2025 were included.

Studies focusing on treatments for the gag reflex in non-dental procedures, as well as those addressing conditions other than gag reflex-related symptoms, were excluded. Animal studies, laboratory research, narrative reviews, systematic reviews, meta-analyses, abstracts, conference proceedings, and non-peer-reviewed publications were also excluded.

A data cleaning process, including the removal of irrelevant keywords (e.g., articles), was conducted before data extraction to enhance accuracy. A predesigned Microsoft Excel spreadsheet was used to collect article data, such as publication year, authors, country/region, affiliations, title, journal, Journal Citation Reports (JCR) division, impact factor (IF), keywords, references, and citations (Mainly in Scopus, WoCC if unavailable). Additionally, data on the participant demographics (sample size, sex, and age), protocols (agent type and relevant details), and outcome indicators (heart rate, pulse, blood pressure, oxygen saturation, and gag reflex status) were extracted.

**Figure 1:**
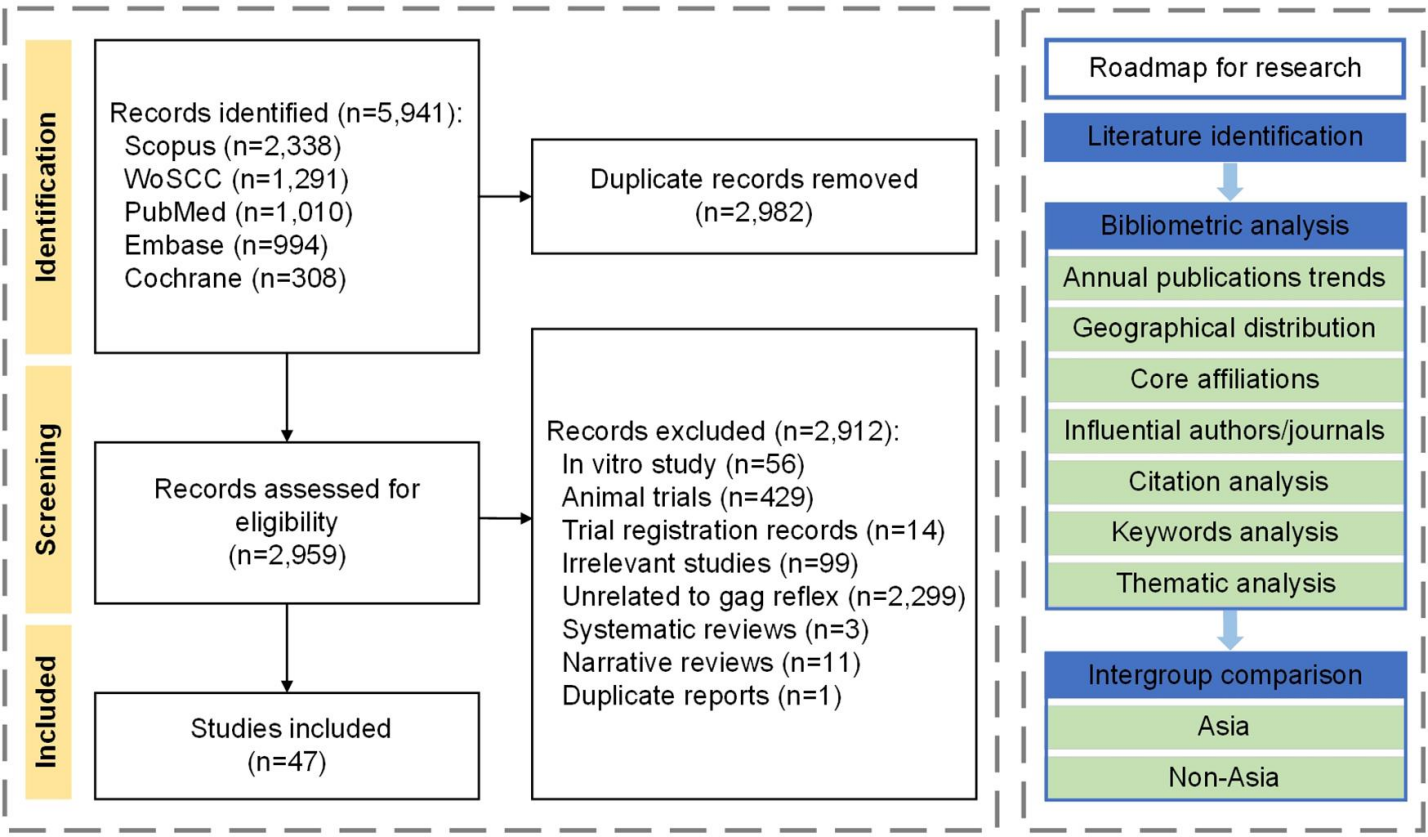
Flowchart of study selection and research methodology.

The annual and global distribution of studies in influential journals, countries, and affiliations, as well as the emerging topic trends, were assessed. Descriptive bibliometric, citation, keyword, and thematic analyses were also performed. Differences in publication years, study designs, sex and age distribution, dental procedures, gag reflex symptoms, interventions, and clinical outcomes between Asian and non-Asian groups were assessed. Bibliographic data were mapped using the bibliometrix and ggplot packages in R software (ver. 4.2.0). The study selection process and methodology are illustrated in the flowchart (Figure 1).

## 3 Results

**Figure 2:**
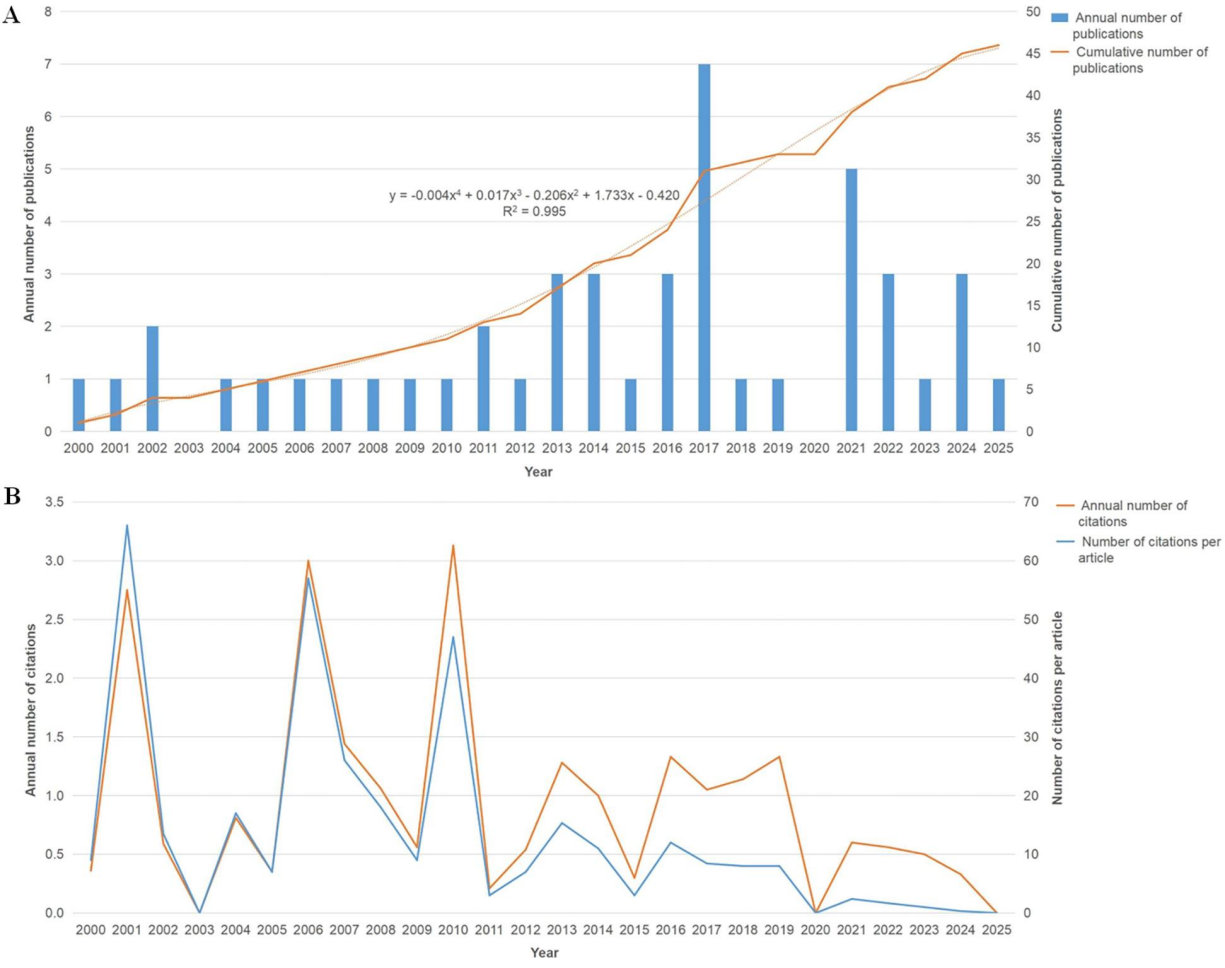
Trends in publication count and citation frequency. **(A)** Annual trends in publication count. **(B)** Annual trends in citation frequency.

A total of 47 studies conducted between 2000 and 2025, which were published in 37 journals and authored by 157 individuals from nine countries, were identified. The cumulative number of publications exhibited an exponential trend $\left(y=-0.004x^{4}+0.017x^{3}-0.206x^{2}+1.733x-0.420,R^{2}=0.995\right)$, with an average annual increase in publications of 4.68% (Figure 2A). The average number of annual citations of most studies ranged from 0 to 3. The data showed a declining trend in citations per article, with a notable decrease from 66 in 2001 to 57 in 2006, reaching zero by 2020 and 2025.

**Figure 3:**
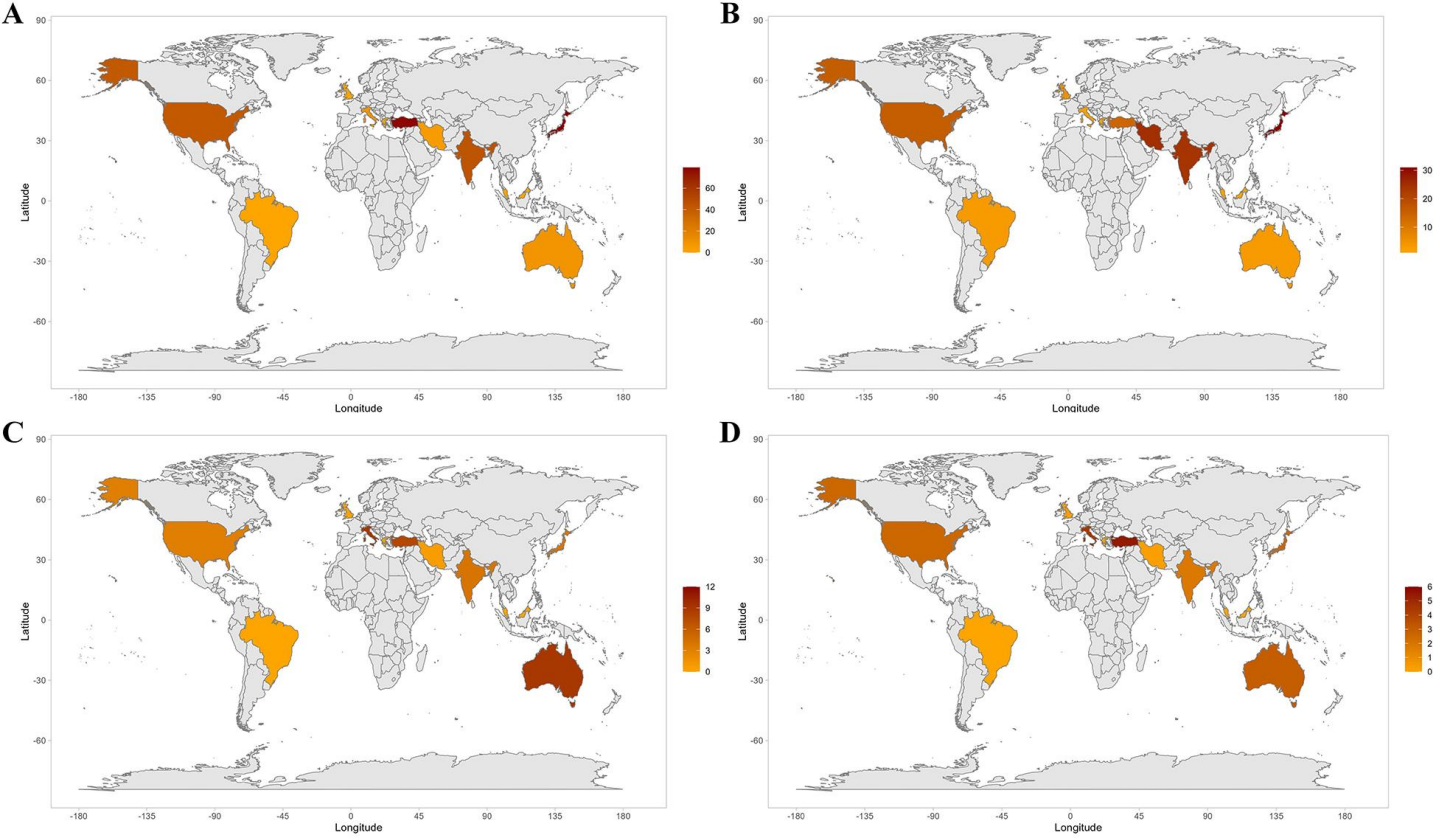
The geographic contribution of countries. **(A)** Overall publications. **(B)** Overall citations. **(C)** Mean citations per year. **(D)** Mean citations per publication.

Five countries contributed a minimum of 10 studies: Japan, Iran, India, USA, and Turkey. Articles from these countries also ranked the highest in citations (Table 1). Studies from Lebanon had the highest citation frequency, with 12 citations per year and 6 citations per article (Figure 3). The institutions with the highest number of publications were Case Western Reserve University (USA), Nippon Dental University School of Life Dentistry at Tokyo (Japan), and Tokyo Dental College (Japan), each publishing six articles.

**Table 1:** Most productive and influential countries, authors, and journals.

Rank	Country productivity (publications)	Country influence (citations)	Author productivity (publications)	Author influence (citations)	Journal productivity (publications)	Journal influence (citations)
1	Japan (31)	Japan (79)	Fiske J. (2)	Fiske J. (123)	British Dental Journal (3)	British Dental Journal (170)
2	Iran (25)	Turkey (77)	Kim S. (2)	Bundgaard M. (57)	Acupuncture in Medicine (1)	Journal of Oral Rehabilitation (36)
3	India (24)	India (44)	Shin S. (2)	Pedersen A.M.L. (57)	Alternative Therapies in Health and Medicine (1)	Tohoku Journal of Experimental Medicine (26)
4	USA (16)	USA (41)	Bundgaard M. (1)	Rosted P. (57)	BMC Complementary Medicine and Therapies (1)	Lasers in Medical Science (22)
5	Turkey (14)	Lebanon (12)	Pedersen A.M.L. (1)	Sari E. (47)	BMC Oral Health (1)	Journal of the American Dental Association (18)
6	UK (5)	Australia (9)	Rosted P. (1)	Sari T. (47)	Forschende Komplementarmedizin (1)	International Journal of Oral & Maxillofacial Implants (17)
7	Australia (3)	Italy (9)	Sari E. (1)	Fukuda K. (36)	International Journal of Oral & Maxillofacial Implants (1)	Acupuncture in Medicine (9)
8	Brazil (3)	Iran (5)	Sari T. (1)	Ichinohe T. (36)	Journal of Evidence-Based Dental Practice (1)	Journal of Oral and Maxillofacial Surgery (9)
9	Italy (2)	UK (1)	Fukuda K. (1)	Koukita Y. (36)	Journal of Oral and Maxillofacial Surgery (1)	Forschende Komplementarmedizin (8)
10	Lebanon (2)	Brazil (0)	Ichinohe T. (1)	Saita N. (36)	Journal of Oral Rehabilitation (1)	Alternative Therapies in Health and Medicine (1)

Most authors (96.82%) contributed to only a single study. The *British Dental Journal* (Q2, IF = 2, 2023), one of the leading journals in the field, published the highest number of studies (*n* = 3) and received the most citations (*n* = 170) (Table 1).

The top ten cited papers (≥15 times) investigated various interventions for managing the gag reflex during dental procedures (Table 2). Low-level laser stimulation targeting acupuncture points, such as Pericardium 6 (PC6, Neiguan) and Conception Vessel 24 (CV24, Chengjiang), significantly reduced the severity of the gag reflex in both children and adults, facilitating procedures such as radiography and dental impressions (14, 18, 22). Acupuncture, including ear acupuncture and CV24 stimulation, also proved effective in controlling the reflex (27). Pharmacological strategies such as intravenous sedation with propofol and midazolam enabled patients with severe gag reflexes to tolerate restorations (28). Hypnotherapy was successfully used to address blood phobia and gagging during tooth extractions (29). These studies highlighted the value of individualized treatment plans that integrate both traditional and modern approaches. Three studies published in the *British Dental Journal* further explored acupuncture and laser stimulation. One study demonstrated the effectiveness of CV24 red-light laser stimulation (3), another combined CV24 laser stimulation with PC6 acupressure (13), and the third evaluated the success of ear acupuncture in managing the gag reflex (6).

**Table 2:** Top 10 studies with citations ≥15.

Study (Reference)	Title	Journal	Citations
Fiske et al. (27)	The role of acupuncture in controlling the gagging reflex using a review of ten cases	British Dental Journal	66
Rosted et al. (13)	The use of acupuncture in controlling the gag reflex in patients requiring an upper alginate impression: an audit	British Dental Journal	57
Sari and Sari (14)	The role of acupuncture in the treatment of orthodontic patients with a gagging reflex: A pilot study	British Dental Journal	47
Saita et al. (3)	Relationship between gagging severity and its management in dentistry	Journal of Oral Rehabilitation	36
Yoshida et al. (28)	Management of exaggerated gag reflex using intravenous sedation in prosthodontic treatment	Tohoku Journal of Experimental Medicine	26
Goel et al. (22)	Effect of Low-level LASER Therapy on P6 Acupoint to Control Gag Reflex in Children: A Clinical Trial	JAMS Journal of Acupuncture and Meridian Studies	24
Elbay et al. (18)	The use of low-level laser therapy for controlling the gag reflex in children during intraoral radiography	Lasers in Medical Science	22
Noble (29)	The management of blood phobia and a hypersensitive gag reflex by hypnotherapy: a case report.	Dental update	18
Scarborough et al. (15)	Altering the gag reflex via a palm pressure point	Journal of the American Dental Association	18
Kubo and Kimura (6)	Implant surgery for a patient with Parkinson's disease controlled by intravenous midazolam: A case report	International Journal of Oral & Maxillofacial Implants	17

The current analysis included 46.81% case reports, 38.30% RCTs, 10.64% case series, and 4.26% cohorts. Of the 1,346 enrolled participants, the sex of 94.28% was disclosed, with 46.02% being male. Among the 47 included studies, 80.85% focused on adults, 12.77% on adolescents, and 4.26% on children. The relevant treatments performed were as follows: 38.30% extractions, 31.91% restorations, 14.89% dental impressions, 8.51% endodontic treatments, 6.38% periapical radiographs, 4.26% denture procedures, and 2.13% orthodontic treatments.

Interventions, including both pharmacological and non-pharmacological approaches, primarily targeted the alleviation of gag reflex-related symptoms. Of these, 74.47% addressed nausea and vomiting, and 25.53% focused on fear and avoidance. The pharmacological treatments used in 25.53% of the studies included agents such as lidocaine (30.77%), propofol (30.77%), and midazolam (15.38%). Non-pharmacological interventions were also prevalent, with acupuncture used in 21.28% of studies, acupressure or laser stimulation in 12.77%, and Transcutaneous Electrical Nerve Stimulation (TENS) in 2.13%. The most commonly targeted acupoint was PC6 (52.38%), followed by CV24 (19.05%) and other acupoints such as Stomach 36 (ST36, Zusanli), and Extra Point 1 (EX1, Jiachengjiang). Additional interventions included hypnotherapy (6.38%), training dentures (6.38%), natural sounds (4.26%), behavioral therapy, earplugs, meditation, nitrous oxide and oxygen, and aromatherapy (e.g., peppermint essential oil). Only 38.30% of the studies employed a placebo. While pharmacological treatments were more frequently studied, non-pharmacological interventions such as acupuncture, acupressure, and laser therapy demonstrated more consistent effectiveness in the available evidence (Table 3).

**Table 3:** Summary of published evidence for different management techniques of the gag reflex.

Management technique	Published studies, *n* (%)	RCTs, *n* (%)	RCTs Reporting Effectiveness, *n* (%)
Drug	13 (27.66)	5 (38.46)	4 (80.00)
Acupuncture	10 (21.28)	3 (30.00)	3 (100.00)
Laser stimulation	6 (12.77)	5 (83.33)	5 (100.00)
Acupressure	6 (12.77)	3 (50.00)	3 (100.00)
Hypnotherapy	4 (8.51)	0 (0.00)	0 (0.00)
Training dentures	3 (6.38)	0 (0.00)	0 (0.00)
Nitrous oxide and oxygen	2 (4.26)	0 (0.00)	0 (0.00)
Distraction techniques	2 (4.26)	1 (50.00)	1 (100.00)
TENS	1 (2.13)	1 (100.00)	1 (100.00)
Natural sounds	1 (2.13)	1 (100.00)	1 (100.00)
Aromatherapy	1 (2.13)	1 (100.00)	1 (100.00)
Behavior therapy	1 (2.13)	0 (0.00)	0 (0.00)

RCT, randomized controlled trial; TENS, transcutaneous electrical nerve stimulation.

Various clinical outcomes were used to assess physiological indicators and gag reflex status. Physiological indicators included oxygen saturation (12.77%), blood pressure (10.64%), pulse rate (10.64%), and heart rate (6.38%). The status of the gag reflex was evaluated using several scales, with the Gagging Severity Index (GSI) being the most frequently used (23.40%), followed by the Gagging Prevention Index (GPI) and the Modified Dental Anxiety Survey (MDAS) (12.77% each). Other assessment tools included the Visual Analog Scale (VAS) (6.38%), the Classification of Gagging Problem Index (CGPI), the Facial Image Scale (FIS), and the Gagging Threshold and Pressure Index (GTPI), each applied in 4.26% of the studies, along with other unlisted tools.

**Figure 4:**
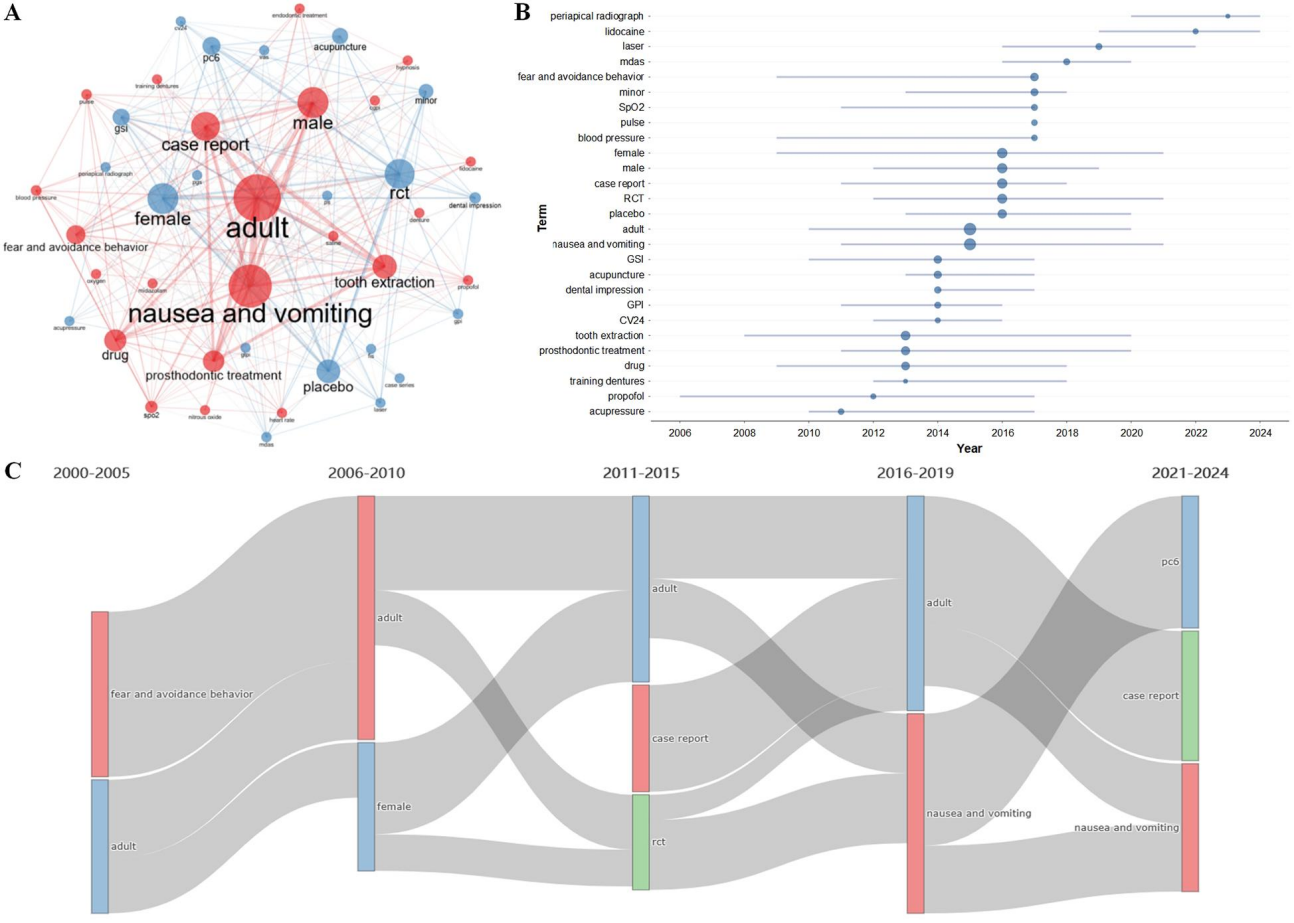
Maps of keywords. **(A)** Keyword co-occurrence map. Node size indicates keyword frequency and line thickness represents co-occurrence frequency. **(B)** Trend topics. Horizontal lines and nodes show the duration and median time of keyword appearances. **(C)** Thematic evolution. Each vertical bar represents keyword prevalence over time, with lines indicating the transition or continuity of terms across periods.

After removing duplicates, 70 unique keywords were identified. Keyword co-occurrence and thematic analyses highlighted the evolving focus of research on the pathophysiology and management of the gag reflex (Figures 4A,B). Early studies (2000–2005) primarily examined adults, focusing on nausea and vomiting, pharmacological agents, and psychological aspects such as fear and avoidance behavior (Figure 4C). Research from to 2006–2010 introduced sex-based differences with an emphasis on female patients and continued pharmacological exploration (Figure 4C). Between 2011 and 2015, a shift toward evidence-based practices emerged, marked by an increase in placebo-controlled studies as well as the adoption of standardized tools such as the GSI and GPI for objective assessment. Case reports during this period focused on gag reflex management in restorations and extractions. From 2016 to 2019, research expanded to include both pharmacological and non-pharmacological therapies, including acupuncture and laser stimulation, with a notable focus on the PC6 acupoint and placebo-controlled trials (Figure 4C). This period also witnessed continued studies on nausea and vomiting, especially in female patients, using tools such as the MDAS. Recent research (2021–2024) has refined methodologies and interventions, emphasizing the clinical importance of gag reflex management in dental practice. The increasing co-occurrence of terms such as placebo, GSI, laser stimulation, and PC6 reflects the growing interest in integrative treatment approaches that combine pharmacological, psychological, and alternative modalities. This evolution reflects a shift from descriptive studies to rigorous clinical trials and interdisciplinary protocols, contributing to the standardization of gag reflex management in clinical settings.

The analysis highlighted significant differences between Asian and non-Asian groups across various dimensions, including publication year, study design, demographic characteristics, dental procedures, gag reflex symptoms, interventions, and clinical outcomes (*P* < 0.05) (Table 4). The mean publication year of the non-Asian group (2,012.00 ± 6.10) was significantly earlier than that of the Asian group (2,016.42 ± 5.97). The Asian group demonstrated a higher proportion of RCT studies (50.00%) than the non-Asian group (23.81%), whereas the non-Asian group exhibited a greater prevalence of case reports (57.14%) and case series (14.29%). Demographic analysis revealed similar sex distributions in both groups, with a slightly higher proportion of females. However, no significant differences in the age distribution were observed. Differences in relevant treatments were observed, with the Asian group performing more periapical radiographs (29.29%) but fewer dental impressions (5.27%) than the non-Asian group. Regarding gag reflex symptoms, both groups exhibited a high prevalence of nausea and vomiting. However, the Asian group had a significantly higher proportion of fear and avoidance behavior (26.66% vs. 15.11%, respectively). In terms of interventions, the Asian group relied more heavily on pharmacological approaches (46.36%), whereas the non-Asian group demonstrated greater use of TENS, nitrous oxide/oxygen, and placebo. Clinical outcomes indicated a higher proportion of favorable results in the Asia group, as reflected by metrics such as oxygen saturation (15.07%), pulse rate (14.96%), blood pressure (8.75%), and heart rate (6.01%), alongside a greater prevalence of outcomes related to the MDAS (15.38%), CGPI (11.70%), FIS (10.12%), Gagging-Related Impression Success Scale (5.69%), Subjective Severity of Gag Reflex (3.16%), Dental Fear Survey (2.53%), and Oral Health Impact Profile (2.11%). Conversely, the non-Asian group demonstrated a higher proportion of reported discomfort, as evidenced by the elevated percentages of GSI (60.71%), VAS (16.88%), numeric rating scale (14.11%), and Predictive Gagging Survey (9.82%).

**Table 4:** Differences in characteristic distribution between Asian and non-Asian groups (*n* = studies/participants).

Characteristics	Asia	Non-Asia	*P*-value
Publication, years	2,016.42 ± 5.97	2,012.00 ± 6.10	0.019*
Study design, *n* (%)			0.328
RCT	13 (50.00)	5 (23.81)	
Case report	10 (38.46)	12 (57.14)	
Case series	2 (7.69)	3 (14.29)	
Cohort	1 (3.85)	1 (4.76)	
Sex, *n* (%)			<0.001*
Female	533 (56.16)	229 (57.68)	
Male	416 (43.84)	168 (42.32)	
Age, *n* (%)			0.446
Adult	763 (80.40)	327 (82.37)	
Pediatric	186 (19.60)	70 (17.63)	
Dental procedure, *n* (%)			<0.001*
Periapical radiograph	278 (29.29)	14 (3.53)	
Dental impression	50 (5.27)	191 (48.11)	
Extraction	20 (2.11)	19 (4.79)	
Restoration	19 (2.00)	17 (4.28)	
Endodontic treatment	2 (0.21)	12 (3.02)	
Orthodontic treatment	0 (0.00)	3 (0.76)	
Denture	0 (0.00)	6 (1.51)	
Gag reflex symptom, *n* (%)			<0.001*
Nausea and vomiting	816 (85.99)	337 (84.89)	
Fear and avoidance behavior	253 (26.66)	60 (15.11)	
Intervention, *n* (%)			<0.001*
Drug	440 (46.36)	4 (1.01)	
Placebo	162 (26.55)	136 (34.26)	
Acupuncture	106 (11.17)	102 (25.69)	
Distraction technique	90 (9.48)	0 (0.00)	
Laser stimulation	61 (6.43)	28 (7.05)	
Acupressure	42 (4.43)	37 (9.32)	
Aromatherapy	24 (2.53)	0 (0.00)	
Natural sounds	20 (2.11)	0 (0.00)	
Training denture	3 (0.32)	0 (0.00)	
Hypnotherapy	1 (0.11)	4 (1.01)	
TENS	0 (0.00)	18 (4.53)	
Nitrous oxide and oxygen	0 (0.00)	17 (4.28)	
Behavior therapy	0 (0.00)	1 (0.25)	
Clinical outcome, *n* (%)			<0.001*
MDAS	146 (15.38)	41 (10.33)	
Oxygen saturation	143 (15.07)	1 (0.25)	
Pulse	142 (14.96)	1 (0.25)	
GPI	111 (11.70)	216 (54.41)	
CGPI	111 (11.70)	0 (0.00)	
FIS	96 (10.12)	0 (0.00)	
GSI	84 (8.85)	241 (60.71)	
Blood pressure	83 (8.75)	1 (0.25)	
Heart rate	57 (6.01)	1 (0.25)	
GISS	54 (5.69)	0 (0.00)	
GTPI	30 (3.16)	41 (10.33)	
PS	30 (3.16)	20 (5.04)	
SSGR	30 (3.16)	0 (0.00)	
DFS	24 (2.53)	0 (0.00)	
GAS	24 (2.53)	0 (0.00)	
OHIP	20 (2.11)	0 (0.00)	
VAS	0 (0.00)	67 (16.88)	
NRS	0 (0.00)	56 (14.11)	
PGS	0 (0.00)	39 (9.82)	
RASS	0 (0.00)	1 (0.25)	

CGPI, Classification of Gagging Problem Index; DFS, Dental Fear Survey; FIS, Facial Imaging Scale; GAS, Gagging Assessment Scale; GPI, Gagging Prevention Index; GISS, Gagging-Related Impression Success Scale; GSI, Gagging Severity Index; GTPI, Gagging Threshold and Pressure Index; MDAS, Modified Dental Anxiety Survey; NRS, Numeric Rating Scale; OHIP, Oral Health Impact Profile; PC, Patient Satisfaction; PGS, Predictive Gagging Survey; RASS, Richmond Agitation-Sedation Scale; RCT, randomized controlled trial; PS, patient satisfaction; SSGR, Subjective Severity of Gag Reflex; TENS, Transcutaneous Electrical Nerve Stimulation; VAS, Visual Analog Scale.

* *p* < 0.05.

## 4 Discussion

The gag reflex is a protective response triggered by stimulation of the soft palate, throat, or mouth to prevent choking or aspiration (3). Although it serves as a protective mechanism, it can interfere with dental treatments and procedures (30). Exaggerated gag reflex sensitivity may be caused by anatomical factors, neurological conditions (e.g., Parkinson's disease, multiple sclerosis), psychological factors (e.g., anxiety, fear), or medical conditions (e.g., gastroesophageal reflux, respiratory infections, postoperative issues). Pharmacological treatments manage the gag reflex by minimizing sensory input and alleviating anxiety, whereas nonpharmacological alternatives are considered for patients who prefer to avoid medications (25, 31). The growth in publications addressing gag reflex management indicates an increased awareness; however, the decrease in citations underscores the necessity for more robust research to improve treatment methods and investigate innovative approaches in dental care.

Japan and Turkey combine traditional therapies, such as acupuncture, with modern dental practices to improve patient comfort and well-being (5, 6, 9, 14, 18, 27, 32–34). In contrast, the USA emphasizes evidence-based, patient-centered care, integrating noninvasive methods such as acupuncture and TENS with pharmacological and behavioral therapies (10, 11, 15, 16, 35–37). Iran focuses on a combination of medications, hypnosis, laser therapy, and acupuncture to prioritize oral health (4, 17, 38–40), while India emphasizes acupuncture, acupressure, and meditation, with an increasing interest in laser therapy for gag reflex management (18, 41–44). The highest publication output in this field originated from institutions in Japan and the USA. A widely cited Lebanese study on intellectual distraction for managing gag reflex and anxiety in children effectively demonstrated the benefits of non-pharmacological interventions in enhancing comfort and reducing gag reflex severity, significantly impacting pediatric dental practice (45). Most authors have contributed to only one publication, indicating the need for greater international collaboration. Extensive publications in the *British Dental Journal* suggest that expanded global cooperation could further enhance the influence of this journal in the field.

The prevalence of RCTs in gag reflex management research emphasizes the importance of high-quality evidence, whereas the absence of cohort studies indicates a gap in the long-term follow-up of individuals with heightened gag reflex sensitivity. Frequent reporting of participants' sex reflects a balanced sex distribution, and the focus on adult populations indicates the relative ease of recruiting adult patients. Ethical considerations and limited treatment options likely contribute to the underrepresentation of children and adolescents in the literature. Effective gag reflex management is particularly crucial in procedures involving deep oral manipulation, such as extractions, restorations, and dental impressions, in which gagging is more likely to be triggered. However, it is less critical in simpler procedures such as routine cleaning. Effective management of the gag reflex during dental procedures involves a blend of pharmacological and non-pharmacological strategies that address both physiological and psychological aspects. Pharmacological options such as lidocaine and propofol offer quick relief, whereas non-pharmacological techniques such as acupuncture are valued for their minimal invasiveness and compatibility with traditional therapies (31). Acupuncture targets specific acupoints to modulate the body's response to the gag reflex and enhance patient comfort. For example, PC6 is selected to reduce nausea and anxiety, CV24 for oral relaxation, ST36 for gastrointestinal distress, and EX1 to calm the throat and jaw (3, 12, 14, 17, 18, 34, 38, 39, 42, 46). Psychological interventions such as hypnotherapy and behavioral therapy targeting dental anxiety have been shown to alleviate the gag reflex (24, 37, 47). However, natural therapies such as placebo, earplugs, meditation, and aromatherapy are less clinically validated and less commonly utilized in clinical practice (8, 12, 14, 15, 17–19, 33, 35, 36, 40, 42, 45, 46).

Current strategies often prioritize addressing physical discomforts, such as nausea and vomiting, and further investigation into psychological aspects, such as dental anxiety, is essential to enhance patient compliance and overall comfort. Monitoring physiological indicators, such as oxygen saturation, blood pressure, pulse rate, and heart rate is critical for assessing the effects of pharmacological interventions, particularly in anxious patients (7, 9–11, 24, 28, 48, 49). Additionally, multiple tools have been used to evaluate objective outcomes, such as the GSI for reflex intensity, GPI for preventive effectiveness, MDAS for anxiety level, and VAS and FIS for discomfort (3, 5, 9, 10, 11, 12–14, 16–19, 37, 43, 45, 46). Integrating these tools enables clinicians to deliver personalized care and improve treatment outcomes. Research on gag reflex management has highlighted its multifactorial nature and the need for personalized, integrative approaches. From 2000 to 2005, studies focused on pharmacological and psychological treatments, recognizing the gag reflex as a response to nausea and anxiety, which spurred the exploration of relaxation and behavioral therapies (6, 27, 29, 33, 47, 50). Between 2006 and 2010, studies on sex-based differences and pharmacological interventions highlighted individual variability and advocated personalized treatment plans (9, 14–16, 29). From 2011 to 2015, the adoption of evidence-based practices and standardized tools, such as the GSI and GPI, facilitated more objective, data-driven approaches (6, 13, 20, 33, 35, 37, 41, 42, 45, 47, 51). Between 2016 and 2019, non-pharmacological therapies, such as acupuncture and laser stimulation, gained prominence, reflecting a shift toward more holistic, patient-centered care (9, 10, 16, 18, 36, 37, 38, 45, 48). Since 2021, research has refined these integrative therapies, emphasizing the combination of noninvasive treatments with pharmacological approaches to reduce reliance on sedation and promote multidisciplinary gag reflex management (4, 5, 7, 17, 23, 35, 39, 42, 43, 52–54). Research in non-Asian regions began earlier with a focus on evidence-based practices and standardized tools. Initial studies relied heavily on case reports and case series owing to limited clinical trial data (10, 11, 13, 16, 25, 34, 36, 37, 47, 50, 51, 53). In contrast, research in Asia has seen a significant surge in recent years driven by advancements in healthcare, increased funding, and a shift toward larger RCTs, emphasizing the need for structured, large-scale studies (3, 4, 6–9, 17, 18, 29, 33, 40, 43, 49, 54). Cultural and regional differences have contributed to these trends, with non-Asian regions historically prioritizing foundational theoretical research, whereas Asia has increasingly embraced integrative and practical clinical trials to address evolving patient needs (10, 12–15, 18, 19, 25, 34–36, 37, 47, 50). These trends reflect a global movement toward more comprehensive, data-driven, and patient-centered approaches for managing the gag reflex.

Managing the gag reflex is crucial in other medical settings, such as endoscopy, gastrointestinal examinations, and surgeries, where deeper oral manipulation often elicits stronger gagging than in dental care (4, 55–58). Pharmacological approaches, including local anesthetics, sedatives, and antiemetics, are commonly used to control the gag reflex. In severe cases, deeper sedation or general anesthesia may be necessary, unlike dental procedures that typically rely on local anesthetics and minimal sedation (6). Non-pharmacological treatments, such as acupuncture, laser stimulation, and behavioral therapy, are being explored to alleviate the gag reflex (42, 59–61). Acupuncture at PC6 has shown promise, although further evidence is required to confirm its effectiveness in non-dental contexts (35, 62, 63).

By employing a multifaceted approach that incorporates both physiological and psychological factors, this study offers a comprehensive review of pharmacological and non-pharmacological interventions for managing the gag reflex. It integrates evidence from diverse regions, including both Asian and non-Asian groups. A key strength of this study is its emphasis on personalized, patient-centered care and the increasing use of noninvasive therapies, such as acupuncture and laser stimulation. However, this study had several limitations. The utilization of keyword-based searches within Title/Abstract introduces a potential for bias, which could affect the comprehensiveness of the review. Despite the increasing volume of literature, many studies, particularly in non-Asian regions, rely on case reports and small-scale research, which limits the generalizability of the findings. Moreover, the absence of long-term cohort studies restricts our understanding of the effectiveness of interventions over time, particularly in individuals with heightened gag reflex sensitivity. Although non-pharmacological treatments such as acupuncture and behavioral therapies show promise, further validation is needed. Additionally, the underrepresentation of children and adolescents in the literature highlights a gap in understanding age-specific interventions, and the reliance on subjective measures of discomfort and anxiety suggests the need for more objective and standardized assessment tools in future research.

## 5 Conclusion

The management of the gag reflex has evolved through the integration of both pharmacological and non-pharmacological treatments, with an increasing emphasis on personalized and holistic approaches. Pharmacological interventions offer quick relief, while non-invasive therapies, such as acupuncture and laser stimulation, demonstrate potential as effective long-term solutions. The trend toward individualized care is particularly prominent in countries such as Japan, Turkey, and the USA, reflecting a broader shift toward patient-centered and comprehensive treatment strategies. However, challenges persist, including the reliance on small-scale studies, insufficient long-term follow-up, and inadequate representation of specific groups, especially children and adolescents. These insights highlight the need for more robust, large-scale research, standardized methodologies, and inclusive strategies to enhance gag reflex management for diverse patient populations.

## Data Availability

The original contributions presented in the study are included in the article/Supplementary Material, further inquiries can be directed to the corresponding author.
