# Supplementary material for: Gag reflex management in dental practice: a bibliometric analysis from 2000 to 2025
Source: Front Oral Health. 2025 Sep 24;6:1666164. doi: 10.3389/froh.2025.1666164 (PMC12504200; doi:10.3389/froh.2025.1666164)
Supplement: Supplementary file 1 [file Table1.docx]

**Additional file 1**

**Table 1** Data sources, search strategy, and outcomes

| Database | Retrieval formula | Results (2025-02-10) |
| --- | --- | --- |
| Scopus | #1 TITLE-ABS-KEY = (gag* OR pharyng* OR retch*)  #2 TITLE-ABS-KEY = (dental OR tooth OR teeth)  #3 LANGUAGE = (English)  #4 DOCTYPE = (Article)  #5 PUBYEAR > 1999 AND PUBYEAR < 2026  #6 #1 and #2 and #3 and #4 and #5 | 2,338 |
| Web of Science Core Collection  (SCI-EXPANDED) | #1 TS = (gag* OR pharyng* OR retch*)  #2 TS = (dental OR tooth OR teeth)  #3 LA = (English)  #4 DT = (Article)  #5 PY = (2000 OR 2001 OR 2002 OR 2003 OR 2004 OR 2005 OR 2006 OR 2007 OR 2008 OR 2009 OR 2010 OR 2011 OR 2012 OR 2013 OR 2014 OR 2015 OR 2016 OR 2017 OR 2018 OR 2019 OR 2020 OR 2021 OR 2022 OR 2023 OR 2024 OR 2025)  #6 #1 and #2 and #3 and #4 and #5 | 1,291 |
| PubMed (MEDLINE) | #1 "gag*"[Title/Abstract] OR "pharyng*"[Title/Abstract] OR "retch*"[Title/Abstract]  #2 "dental"[Title/Abstract] OR "tooth"[Title/Abstract] OR "teeth"[Title/Abstract]  #3 English[Language]  #4 Publication data: from 2000 to 2025  #5 #1 and #2 and #3 and #4 | 1,010 |
| CENTRAL (The Cochrane Library) | #1 (gag* OR pharyng* OR retch*):ti,ab,kw  #2 (dental OR tooth OR teeth):ti,ab,kw  #3 Publication data: from 01/01/2000 to 06/02/2025  #4 #1 and #2 and #3 | 308 |
| Embase | #1 gag*:ti,ab,kw OR pharyng*:ti,ab,kw OR retch*:ti,ab,kw  #2 dental:ti,ab,kw OR tooth:ti,ab,kw OR teeth:ti,ab,kw  #3 [english]/lim  #4 [article]/lim  #5 2000:py OR 2001:py OR 2002:py OR 2003:py OR 2004:py OR 2005:py OR 2006:py OR 2007:py OR 2008:py OR 2009:py OR 2010:py OR 2011:py OR 2012:py OR 2013:py OR 2014:py OR 2015:py OR 2016:py OR 2017:py OR 2018:py OR 2019:py OR 2020:py OR 2021:py OR 2022:py OR 2023:py OR 2024:py OR 2025:py  #6 #1 and #2 and #3 and #4 and #5 | 994 |
